# A mobile hybrid deep learning approach for classifying 3D-like representations of Amazonian lizards

**DOI:** 10.3389/frai.2025.1524380

**Published:** 2025-08-12

**Authors:** Arthur Gonsales da Silva, Roger Pinho de Oliveira, Caio de Oliveira Bastos, Elena Almeida de Carvalho, Bruno Duarte Gomes

**Affiliations:** ^1^Departamento de Ciência de Dados, Instituto Tecnológico Vale, Belém, Brazil; ^2^Centro de Ciências Biológicas e da Saúde, Universidade da Amazônia, Belém, Brazil; ^3^Instituto de Ciências Biológicas, Universidade Federal do Pará, Belém, Brazil

**Keywords:** hybrid machine learning, 3D representations, Amazonian lizards, MobileNet, species classification

## Abstract

Image classification is a highly significant field in machine learning (ML), especially when applied to address longstanding and challenging issues in the biological sciences, such as specie recognition and biodiversity conservation. In this study, we present the development of a hybrid machine learning-based tool suitable for deployment on mobile devices. This tool is aimed at processing and classifying three-dimensional samples of endemic lizard species from the Amazon rainforest. The dataset used in our experiment was collected at the Museu Paraense Emílio Goeldi (MPEG), Belém-PA, Brazil, and comprises three species: (a) *Anolis fuscoauratus*; (b) *Hoplocercus spinosus*; and (c) *Polychrus marmoratus*. We compared the effectiveness of four artificial neural networks (ANN) for feature extraction: (a) MobileNet; (b) MobileNetV2; (c) MobileNetV3-Small; and (d) MobileNetV3-Large. Additionally, we evaluated five classical ML models for classifying the extracted patterns: (a) Support Vector Machine (SVM); (b) GaussianNB (GNB); (c) AdaBoost (ADB); (d) K-Nearest Neighbors (KNN); and (e) Random Forest (RF). The performance metrics of all classifiers were very close, we used the McNemar’s test on each model’s confusion matrix to evaluate and compare their statistical significance. Our best model was a combination of a 2.9 million parameters MobileNetV3-Small as the feature extractor, with a linear kernel-based SVM as the classifier, which achieved accuracy of 0.955, precision of 0.948, recall of 0.948, and f1-score of 0.948. The results indicated that the use of a small deep learning (DL) model, in combination with a classical ML algorithm, emerges as a viable technique for classifying three-dimensional representations of lizard species samples. Such an approach facilitates taxonomic identification work for professionals in the field and provides a tool adaptable for integration into mobile data recording equipment, such as smartphones, and benefiting from more morphological features extracted from three-dimensional samples instead of two-dimensional images.

## 1 Introduction

In the Squamata order, which comprises species with bodies covered by scales, among other characteristics, the classification of lizards is based on multiple morphological features (Pyron et al., 2013). According to (Stewart and Daniel, 1075), these morphological characteristics are referred to as microornamentations and are most prominent in the dorsal scales of the head, trunk, and tails of each individual. Modern biodiversity data collection equipment, such as sound recorders, camera traps, and other imaging methods, allow the measurement of many parameters, making it possible to extract vast amounts of information in a relatively inexpensive manner. This technology has become increasingly popular among scientists and helps to answer questions such as: (a) Which species occur in a given area?; (b) What are their activities/behavior?; and (c) How many individuals inhabit the region? (Gomez Villa et al., 2017). The success in inventorying and monitoring forest lizard species relies on robust monitoring, recognition, and sampling, and currently represents one of the most complex tasks in the field of herpetological conservation (Bell, 2009).

One of the most used data types in problems involving biodiversity conservation with specialized image models is camera trap images (Miao et al., 2019). The aim of remote monitoring can range from species identification to inferring the abundance and distribution of important conservation animals, but these motivations typically share a common goal: to classify target species (Chen et al., 2019). This interest in remote monitoring is accompanied by several challenges in large-scale identification (Chen et al., 2019).

The most recent research in automated identification of animal species can be divided into two distinct types: laboratory-based investigation (LBI), and field-based investigation (FBI) (Martineau et al., 2017). For LBI, a pre-established image acquisition protocol must be followed to standardize the sampling and use of specimens, which are typically handled by a specialized biologist. This contrasts significantly with FBI, where a mobile device or camera is usually employed for the image acquisition process of the individuals (Martineau et al., 2017).

In studies of insect classification, for instance, LBI is the most commonly used method due to the highly manual handling of specimens (Martineau et al., 2017). On the other hand, the identification of mammals and fish is typically accomplished using field-recorded images, while automated recognition of plant species can benefit from both the controlled environment of a laboratory and field conditions (Weinstein, 2018). These studies focus on the use of Machine Learning (ML) with Convolutional Neural Networks (CNN), which are models specialized in image processing that extract high-level abstractions from data and are considered the state-of-the-art for tasks involving image classification (Wäldchen and Mäder, 2018).

The most common type of algorithm learning used for image classification is supervised learning, where input data (samples) are fed into the model along with their corresponding labels (class names), and the algorithms are trained to map the input information to the output label, such as the name of a species, for example (Norouzzadeh et al., 2021).

Before the emergence of computer vision (CV) models and artificial intelligence (AI) algorithms in general, the process of identifying and conserving animal species was and still is, in some places, carried out manually with a high dependence on human activities, which imposes several limitations on the task (Tuia et al., 2022). These limitations, mainly physical and cognitive, hinder the understanding of species distribution and diversity. For instance, the counting of colonies of seabirds and cave-dwelling bats conducted by humans tends to significantly underestimate the actual number of individuals (Tuia et al., 2022). This scenario of limitations and uncertainties changed with the advent of large-scale AI-driven automation of these tasks.

With recent advances in automated image classification and information gathering, new approaches have become possible (Pinho et al., 2023). Several existing examples demonstrate the applications of automatic classification based on deep learning (DL) using taxonomic data from different species (Wäldchen and Mäder, 2018). Table 1 summarizes recent studies where CV algorithms were employed to perform automated species identification on a diverse range of other taxonomic datasets (Weinstein, 2018; Tuia et al., 2022; Bolon et al., 2022; Durso et al., 2021; Binta Islam et al., 2023).

**Table 1:** Recent studies employing computer vision algorithms for species classification across various taxonomic groups.

Species	Samples	Architecture	Accuracy	Study
Reptiles	386,006	Vision Transformer (ViT)	0.962	Bolon et al. (2022)
Reptiles	82,601	EfficientNet	0.870	Durso et al. (2021)
Lizards & Amphibians	6,045	MobileNetV2	0.820	Gill et al. (2024)
Lizards & Amphibians	2,700	VGG16	0.870	Binta Islam et al. (2023)
Fishes	1,080	Image Processing + SVM	0.942	Sharmin et al. (2019)
Fishes	3,068	U-NET + CNN	0.979	Robillard et al. (2023)
Lizards & Amphibians	828	CNN	0.600	Islam and Valles (2020)
Mammals	326	Mask R-CNN + ResNet101	0.980	Gray et al. (2019)

A total of seven comparable studies where pre-trained state-of-the-art deep learning models were employed on taxonomic datasets of different groups were analyzed in order to provide more robustness to our research. The sample size and accuracy score are reported below for each study.

As can be seen in Table 1, most studies used pre-trained models. This is the case because when pre-trained networks are employed either as feature extractors or efficiently optimized for the new dataset, there exists a strong correlation between the high accuracy achieved by the model on its original pre-training phases with its score in the new training demand (Kornblith et al., 2019). Thus, incremental or transfer learning only requires the pre-trained model to generalize an additional predictive pattern that might be present in the dataset while retaining its previous optimal weights, often gathered on ImageNet Large-Scale Visual Recognition Competition (ILSVRC) (Durso et al., 2021).

Despite the widespread use of CNNs in taxonomic databases (Weinstein, 2018; Tuia et al., 2022; Bolon et al., 2022; Durso et al., 2021; Binta Islam et al., 2023), our literature review revealed no applications of these models to three-dimensional representations of Amazonian lizards. In this study, we have developed an open-source system for the automatic classification of three-dimensional samples of Amazonian lizard species, adaptable for deployment on mobile equipment such as smartphones. We employed state-of-the-art DL and ML techniques for image processing and classification using the family of CNNs known as MobileNets (Howard et al., 2017; Sandler et al., 2018; Howard et al., 2019), together with classical ML models, which demonstrated exceptional efficiency in similar tasks. Making use of 3D representations of specimens as samples turned our approach unique, and significantly benefited our models with relevant morphological information about each species when compared to typical 2D representations as employed by Islam and Valles (2020), which used the same family of pre-trained CNNs we used in this study, but achieved less accuracy.

## 2 Materials and methods

### 2.1 Data collection and sample processing

**Figure 1:**
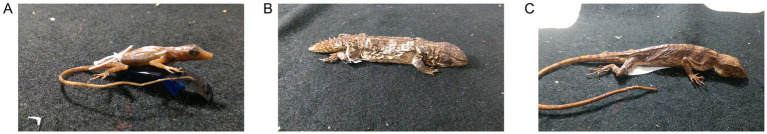
The three species selected for this study. **(A)** *Anolis fuscoauratus*, **(B)** *Hoplocercus spinosus*, **(C)** *Polychurs marmoratus*. All the specimens were preserved in alcohol, and only individuals with good preservation conditions were selected.

Data was collected at MPEG, located in Belém, Para, Brazil. MPEG is the second-oldest scientific research institution in Brazil, founded in 1866, and it houses a local herpetological collection with approximately 100,000 specimens of amphibians and reptiles (Da Costa Prudente et al., 2019). Three species were selected for collection, namely: (a) *Anolis fuscoauratus*; (b) *Hoplocercus spinosus*; and (c) *Polychrus marmoratus*; all species found in the Amazon region (Vitt et al., 2003; Torres-Carvajal and De Queiroz, 2009; Murphy et al., 2017). Figure 1 below shows pictures of individuals from each species.

All specimens were preserved in alcohol, and the preservation conditions of each sample were a determining factor in selecting both the individuals and species chosen for this study. The selected individuals were then placed on a black cloth, and positioned on the collection bench to mitigate any visual noise that could interfere with identification. This simple strategy can be easily replicated in any environment, as in field data collection routines.

In recent studies using three-dimensional samples for species classification, the use of Light Detection and Ranging (LiDAR), and Spectral Imaging (SI) are extensive, particularly in studies using plants as specimens (Mäyrä et al., 2021; Nezami et al., 2020; Polonen et al., 2018). However, these technologies are costly and require highly specialized expertise, making them impractical for everyday use by experts in both laboratory and field settings. Furthermore, using impractical solutions such as LiDAR and SI makes it almost impossible to safely and easily reproduce the results, especially in areas where research funding is unstable.

**Figure 2:**
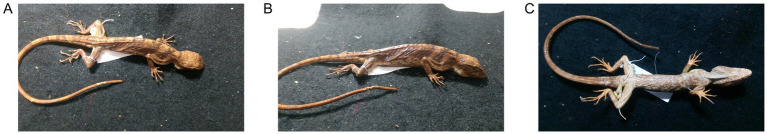
A sample of *Anolis fuscoauratus*, composed of three perspectives. **(A)** dorsal, **(B)** lateral, **(C)** ventral views. The images are converted to grayscale and then arranged into a matrix of dimensions 1 x 224 x 224 x 3, with each image occupying one color channel.

As a solution, we adopted smartphone-based image capture from the dorsal, lateral, and ventral points of view to compose our samples. The use of smartphones offers a cost-effective alternative, enabling broader accessibility and usability for species classification. As can be seen in Figure 2, three photos of each individual were taken, where each will represent one channel of a final RGB-like three-dimensional sample.

It was necessary to remove some images due to poor quality; a total of 80 three-dimensional samples, totaling 240 unique images, remained. Among these, there were 49 samples of *Anolis fuscoauratus*, 22 samples of *Hoplocercus spinosus*, and 9 samples of *Polychrus marmoratus*.

Subsequently, all samples were resized to dimensions of 224 × 224 pixels and standardized to conform to the input layer requirements of our Convolutional Neural Network (CNN), which are standard for the MobileNet family of models. The dataset was then partitioned into training/validation and test sets, adhering to an 80–20% split, respectively. This approach was chosen over the inclusion of an additional hold-out validation set, with a preference for employing cross-validation. The Figure 2 below shows one sample composed of different perspectives.

### 2.2 Data augmentation for addressing class imbalance

**Figure 3:**
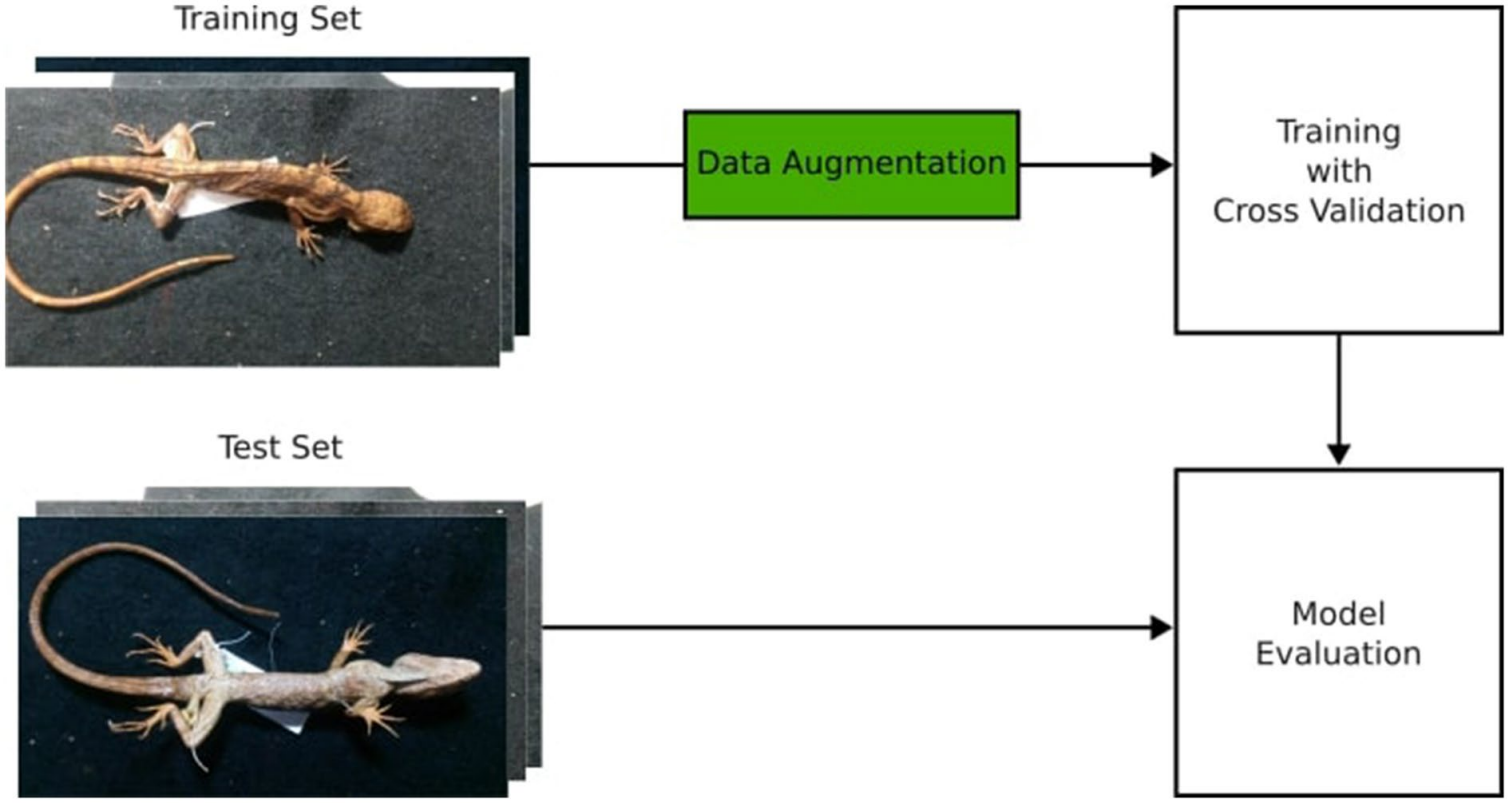
The data augmentation process illustrated. The original image set was split into train and test sets, and then the augmented images were generated for the training set. No images from the test set were used to augment data in the training set.

We used TensorFlow’s (TF) image data generator module (Abadi et al., 2016) for data augmentation, where random modifications such as Flip, Crop, and Translate, were applied to the samples without altering their fundamental characteristics, thus generating new synthetic observations in our dataset (Xu et al., 2023). The outcome of data augmentation resulted in an increase from 80 initial three-dimensional samples to 3,900 in the training set, balanced between species. This increases the robustness of our model on handling different imaging conditions in different collection environments. The Figure 3 illustrates the data augmentation process.

### 2.3 Models selection and definition

We selected the class of MobileNet models for developing our species identification system. This class consists of highly efficient algorithms for mobile CV applications and embedded systems (Howard et al., 2017). There are three main MobileNet models: (a) MobileNet; (b) MobileNetv2; and (c) MobileNetV3, with the latter having two variants, namely: Large and Small (Howard et al., 2017; Sandler et al., 2018; Howard et al., 2019).

The first model (MobileNet) is based on depth wise separable convolutions, which are a form of factorized convolutions that transform a regular convolution operation into depth wise, which significantly reduces both computational cost and model size, having 4.3 M adjustable parameters, with a lower memory footprint in comparison to other major CNNs (Howard et al., 2017). The second model (MobileNetV2) introduces the new *inverted residual with a linear bottleneck* module (Sandler et al., 2018), which expands to a higher dimension a compressed low-dimensional representation of the input data and then filters it using a lightweight depthwise convolution, having a slightly higher memory requirement than MobileNet, with a more robust architecture comprised of 3.5 M adjustable parameters. The third model (MobileNetV3) features an efficient redesign of the network architecture, coupled with a segmentation decoder that optimizes resource consumption for both of its variants, the Large, for devices with greater availability of resources, having 5.4 M adjustable parameters, and the Small, for scenarios with more limited processing power, having a total of 2.5 M adjustable parameters (Howard et al., 2019).

We used and compared the performance of all available MobileNet network variants as feature extractors only. We did not retrain the models, and we appended a Global Average Pooling 2D layer at the end of each model for dimensionality reduction, and then we replaced their classification layers with classical ML algorithms.

The selection of classical ML algorithms was based on the criteria that it has to be commonly applied in research with biological databases (Jovel and Greiner, 2021), and pre-implemented in Scikit-learn (SKL) (Pedregosa et al., 2008). The chosen models were: (a) Support Vector Machine (SVM) with linear, rbf, poly kernels; (b) K-Nearest Neighbors (KNN); (c) Random Forest (RF); (d) GaussianNB (GNB); and (e) AdaBoost (ADB).

We adopted this hybrid approach because there is enough evidence showing that using pre-trained models, such as MobileNets as feature extractors, can transfer their high accuracies acquired on ILSVRC to the new models they compose, without the need for computationally expensive retraining (Kornblith et al., 2019; Sowmya et al., 2023; Michele et al., 2019). Moreover, the composition of a hybrid model with a classical algorithm serving as the final classifier drastically reduces the likelihood of the model presents overfitting (Michele et al., 2019).

### 2.4 Feature extraction and dimensionality reduction

From the original data, we generated four new datasets of features, each one extracted with a different variant of MobileNet (V1, V2, V3-Large, and V3-Small), we call these full-features datasets. To assess the complexity and the effectiveness of feature separation across our classes, we applied the t-distributed Stochastic Neighbor Embedding (t-SNE) to each of the full-feature datasets. t-SNE is a method that compresses high-dimensional data into a two-or three-dimensional map (van der Maaten and Hinton, 2008), effectively transforming high cardinality information into a lower-dimensional compressed space.

Lastly, we used the RF algorithm to ascertain the relative importance of features within each full-features dataset (Haq et al., 2019). Subsequently, a significance threshold of 0.01 was applied to retain only those features ranking highest in importance. This process yielded a subset of 20 columns constituting the top-ranked features for each respective full-features dataset.

### 2.5 Model training and evaluation

For comparison, we trained our ML models on each full-features dataset, and also on each 20 top-ranked features dataset. All datasets were normalized with *MinMaxScaler* (Raju et al., 2020). The training was cross-validated, with the *k-fold* and *random state* parameters set to 4, and 42, respectively. For models’ performance evaluation, we used a total of five different metrics, namely: (a) accuracy; (b) precision; (c) recall; (d) f1-score; and (e) confusion matrix.

#### 2.5.1 Accuracy

Accuracy denotes the ratio of true positives (TP) and true negatives (TN), against the overall predictions, also comprised of false positives (FP) and false negatives (FN) (Naser and Alavi, 2023). It is calculated as follows:

$$\text{Accuracy}=\frac{\mathrm{TP}+\mathrm{TN}}{\mathrm{TP}+\mathrm{TN}+\mathrm{FP}+\mathrm{FN}}$$

#### 2.5.2 Precision

Precision denotes the ratio of correctly predicted true instances over the total number of positively predicted instances (Naser and Alavi, 2023). It is calculated as follows:

$$\text{Precision}=\frac{\mathrm{TP}}{\mathrm{TP}+\mathrm{FP}}$$

#### 2.5.3 Recall

Recall denotes the ratio of correctly predicted true instances over the total number of positive instances (Naser and Alavi, 2023). It is calculated as follows:

$$\text{Recall}=\frac{\mathrm{TP}}{\mathrm{TP}+\mathrm{FN}}$$

#### 2.5.4 F1-score

F1-Score is the harmonic mean of precision and recall (Naser and Alavi, 2023). It is calculated as follows:

$$F1-\text{Score}=2x\frac{\text{PrecisionxRecall}}{\text{Precision}+\text{Recall}}$$

#### 2.5.5 Confusion matrix

A confusion matrix presents a summary of correctly and misclassified samples of a classification problem (Naser and Alavi, 2023). The entries of a confusion matrix are all the positive and negative predictions described so far.

### 2.6 Bayesian optimization evaluation

We made an additional evaluation using Bayesian Optimization (BO) in an attempt to further improve the best ML model’s hyperparameters. By using BO, a surrogate for the model’s objective function is created, and a Gaussian Regressor quantifies the uncertainty for the surrogate (Frazier, 2018). The formula below shows the acquisition function Expected Improvement (EI), adopted in this study.

$${\mathrm{EI}}_{n}(x)≔E_{n}$$

The EI tells us how much we expect to improve our best result if we try a new set of optimizable parameters *x*, and it is popular due to its multi-modal nature and effective balance between exploration and exploitation of the search space for the best set of hyperparameters that will produce the lowest error on the model (Wang et al., 2017). The metrics resulting from this attempt were compared to the model trained without the help of BO.

### 2.7 Statistical analysis

The McNemar’s test is a statistical test particularly suitable for comparing the performances of two classification models on the same dataset, assuming a null hypothesis (H0) of no statistical difference between the two proportions being compared. The test was used to evaluate both model performance and the effectiveness of BO throughout this study.

First, we assessed potential performance differences between the best-performing models trained with the full-feature dataset, both with and without BO. This process was then repeated for the best models trained on the reduced dataset (top 20 features), again comparing models with and without BO.

Finally, McNemar’s test was used to determine if there were any significant differences between the best models trained with the full-feature and reduced datasets, regardless of the use of BO during training.

### 2.8 Classification pipeline technologies

Our open-source pipeline was developed using Python (Rossum, 1995), the TF DL framework (Abadi et al., 2016), and the SKL ML framework (Pedregosa et al., 2008). Images were captured using an HTC One M8 smartphone (4MP × 2688 × 1520 440 ppi camera). The classification pipeline comprises five main stages:

- Capture a dorsal photo of the specimen.
- Capture a lateral photo of the specimen.
- Capture a ventral photo of the specimen.
- Compose a three-dimensional sample from the acquired images.
- Classify the lizard species with our trained model.

## 3 Results

### 3.1 Datasets complexity analysis

**Figure 4:**
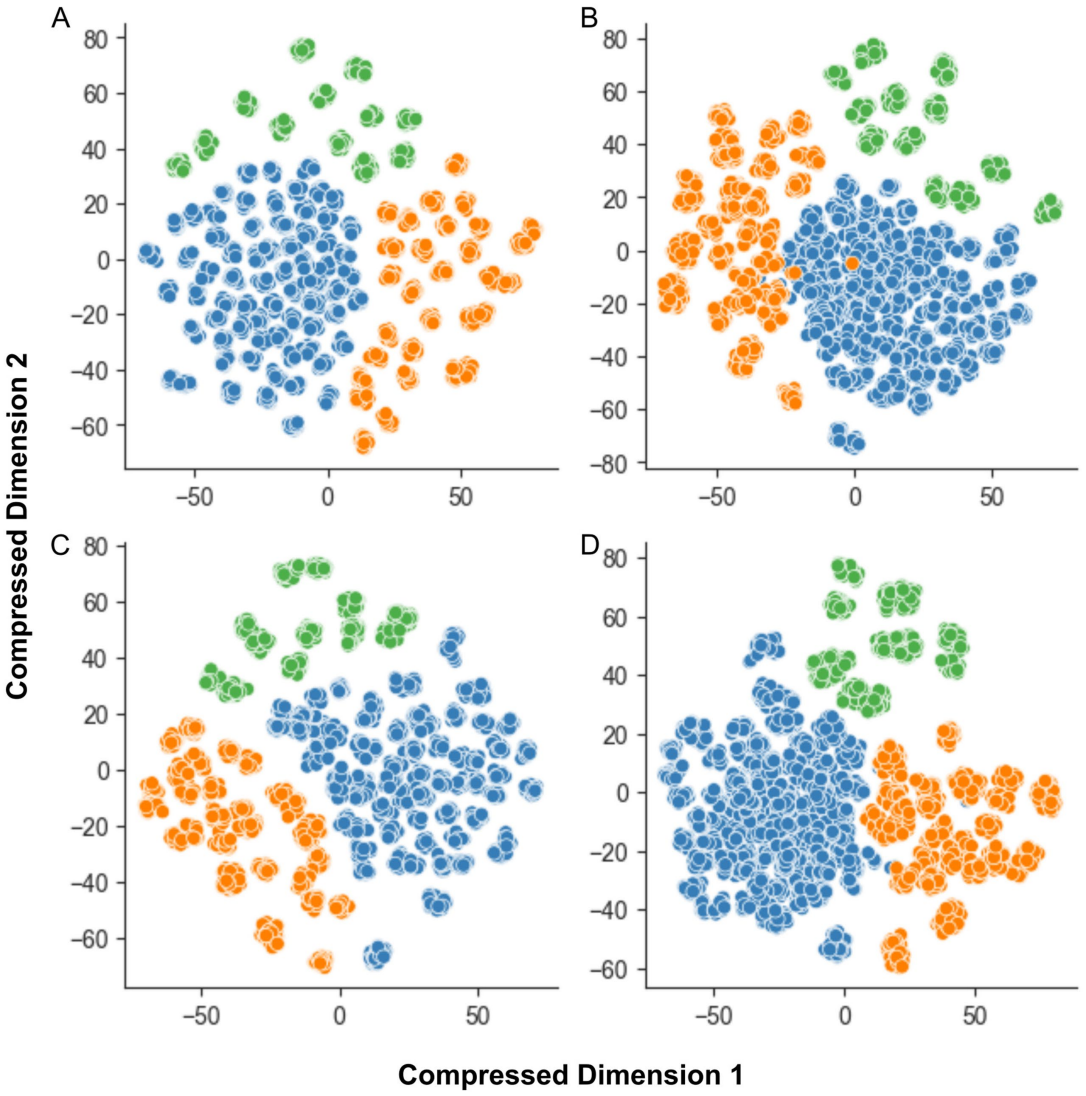
The t-SNE plot for dataset complexity analysis. **(A)** MobileNet; **(B)** MobileNet V2; **(C)** MobileNet V3-Large; and **(D)** MobileNet V3-Small. The features extracted by MobileNet V3-Small demonstrate the most homogeneous and well-separated clusters of data points for all species. The X and Y axes represent compressed dimensions.

The complexity of each dataset significantly influenced the performance of classical ML algorithms. Figure 4 illustrates the differences in clustering for each dataset, as revealed by t-SNE (van der Maaten and Hinton, 2008).

Analyses (a) and (c) show good separation between clusters, but the samples within each cluster are more dispersed. In contrast, analysis (b) reveals greater class overlap, although clusters are relatively well-concentrated. Analysis (d), based on MobileNetV3-Small-extracted data, demonstrates the optimal balance between cluster separation and sample concentration, with minimal class overlap.

### 3.2 Model performance analysis

The trained models demonstrated similar performance across all datasets, indicating that both full-feature and reduced datasets successfully captured essential morphological and structural patterns, such as microornamentations (Stewart and Daniel, 1075). This facilitated model generalization despite variations in the number of extracted features. Table 2 presents the top-performing models trained with cross-validation using all features extracted by MobileNet variants.

**Table 2:** Cross-validated average performance metrics of classic ML models on each full-features dataset.

Feature Extractor	Best model	Accuracy	Precision	Recall	F1-score
MobileNetV3-Small	Linear SVM	0.974	0.985	0.965	0.973
MobileNetV1	Linear SVM	0.970	0.981	0.949	0.961
MobileNetV2	Linear SVM	0.951	0.964	0.924	0.937
MobileNetV3-Large	Linear SVM	0.953	0.969	0.928	0.942

Four models acting as feature extractors were tested together with five different classical machine learning algorithms acting as classifiers. The accuracy, precision, recall, and f1-score for all hybrid models, on the full-features dataset, are reported below.

The MobileNet V3-Small + Linear SVM classifier consistently outperformed other models on full-feature datasets. This superior performance might be attributed to the relatively lower complexity and clearer class separation of the dataset generated with this MobileNet variant, as evidenced by Figure 4. Other datasets exhibited greater class overlap and less cluster concentration.

While the dataset with only the 20 top-ranked features exhibited reduced homogeneity and class separation, it remained representative of the underlying data. Notably, the MobileNet V3-Small + Linear SVM classifier again demonstrated comparable performance, leading the results on this reduced dataset as well as shown in Table 3.

**Table 3:** Cross-validated average performance metrics of classic ML models on each 20 top-ranked features dataset.

Feature Extractor	Best model	Accuracy	Precision	Recall	F1-score
MobileNetV3-Small	Linear SVM	0.968	0.970	0.962	0.965
MobileNetV1	RBF SVM	0.958	0.955	0.936	0.944
MobileNetV3-Large	RFC	0.935	0.934	0.923	0.925
MobileNetV2	Linear SVM	0.898	0.905	0.859	0.874

Four models acting as feature extractors were tested together with five different classical machine learning algorithms acting as classifiers. The accuracy, precision, recall, and f1-score for all hybrid models, on the 20 top-ranked features dataset, are reported below.

The reduced dataset saw more complex classical ML algorithms among the top performers compared to the full-feature dataset (Table 2). This suggests a need for increased model complexity to compensate for the information loss resulting from feature selection.

### 3.3 Bayesian optimization effectiveness analysis and model skill evaluation

McNemar’s test was used to assess statistical differences between hybrid models trained with and without Bayesian Optimization (BO), using both full-feature and reduced datasets.

For the full-feature dataset, the model trained without BO significantly outperformed the BO-trained model (χ^2^ = 0.0, *p* = 3.05e-5), achieving an accuracy of 0.991, precision of 0.987, recall of 0.992, and an F1-score of 0.990 on the test set.

In contrast, for the reduced dataset, the BO-trained model showed superior performance (χ^2^ = 14.0, *p* = 8.58e-11), with an accuracy of 0.955, precision of 0.948, recall of 0.948, and an F1-score of 0.948.

**Figure 5:**
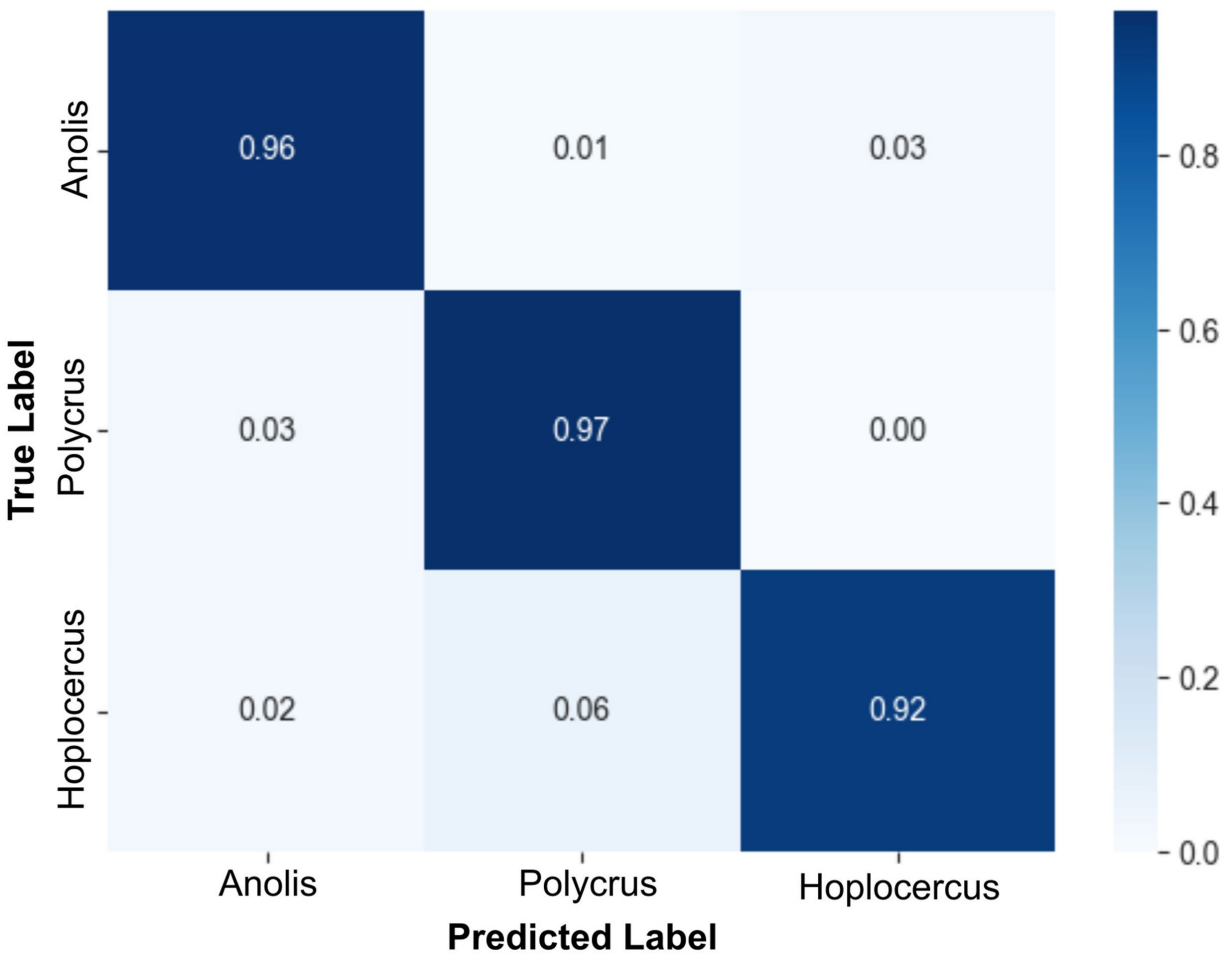
Confusion matrix of the MobileNetV3-Small + Linear SVM model trained with the reduced dataset. This confusion matrix corresponds to the best-performing MobileNetV3-Small + Linear SVM model trained on the dataset with the 20 top-ranked features. Despite the highest number of misclassified samples being from the Polychrus species among all classes, the overall performance, on a per-sample basis, was proven to be highly efficient.

Finally, McNemar’s test revealed no significant difference between the best-performing full-feature model and the best-performing reduced-feature model (χ^2^ = 7.0, *p* = 0.80361). Thus, the less complex model trained with the reduced dataset can be safely used. Figure 5 shows the normalized confusion matrix for the MobileNetV3-Small + Linear SVM model on the test set.

### 3.4 Classification pipeline trainable parameters

The Table 4 summarizes the trainable parameters of the final classification pipeline, which includes a Min-Max scaler and a linear kernel SVM.

**Table 4:** Trainable parameters of the assembled final classification pipeline.

Parameter	Value
minmax_rescaler__clip	False
minmax_rescaler__copy	True
minmax_rescaler__feature_range	(0, 1)
linear_svm_classifier__C	0.10284379327993369
linear_svm_classifier__class_weight	None
linear_svm_classifier__dual	False
linear_svm_classifier__fit_intercept	True
linear_svm_classifier__intercept_scaling	1
linear_svm_classifier__loss	squared_hinge
linear_svm_classifier__max_iter	1,000
linear_svm_classifier__multi_class	‘ovr’
linear_svm_classifier__penalty	‘l2’
linear_svm_classifier__random_state	42
linear_svm_classifier__tol	0.0001

All the trainable model parameters for the classification algorithms are listed below to complete reproducibility of this study.

## 4 Discussion

This research sought to evaluate the efficacy of classifying three-dimensional representations of Amazonian lizard species using cutting-edge deep learning algorithms. The aim was to create a mobile-ready classification pipeline that could be integrated into biodiversity monitoring equipment. The use of image triplets, each containing dorsal, lateral, and ventral views of the specimens, is a distinctive approach compared to all the most recent and comparable studies. Our findings demonstrate that this approach is not only feasible but also an efficient means of automated classification. Furthermore, our unique dataset, collected at MPEG, one of Brazil’s oldest and most renowned research institutions, sets this study apart from a substantial portion of recent research.

### 4.1 Comparative analysis with existing research

Although several studies have applied deep learning to reptile images (Weinstein, 2018; Tuia et al., 2022; Bolon et al., 2022; Durso et al., 2021; Binta Islam et al., 2023), most focus on a broader scope of reptiles and amphibians, not specifically lizards (Binta Islam et al., 2023; Sharmin et al., 2019; Gill et al., 2024). A notable exception is (Gill et al., 2024), which used MobileNetV2 to classify an open-access dataset of reptiles and amphibians, including lizards as one class. Unlike our approach, they treated each image independently, without aggregating triplets. Despite a larger dataset for fine-tuning, their accuracy of 0.820 was significantly lower than our best model. This discrepancy might be due to their higher number of classes, potentially increasing the model’s learning difficulty. However, our dataset arguably presents higher complexity due to variations in dorsal, lateral, and ventral points-of-view, which may have forced our models to learn more detailed morphological patterns. Thus, our use of 3D representations and image triplets might be advantageous for capturing such details, ultimately leading to improved classification performance.

## 5 Conclusion

Our study elucidates the potential for the classification of three-dimensional representations of lizard species through the utilization of mobile-ready deep learning models in the context of biodiversity monitoring. The deployment of three-dimensional representations of the specimens, generated from image triplets comprising dorsal, lateral, and ventral perspectives of the animals, has proven efficacious in capturing intricate morphological patterns. This approach facilitates robust feature extraction, distinct class separation, and enhanced classification accuracy. The capacity of the model to be readily deployed on mobile devices further augments its potential for field applications in biodiversity research and conservation endeavors.

Future research initiatives should focus on augmenting the number of supported species, as well as assessing the impact of this increment in our model of choice, potentially exploring new models and architectures, thereby contributing to the burgeoning field of deep learning-based lizard species classification. Currently, there is a paucity of extensive published studies in this domain for direct comparison. Additionally, efforts to incorporate a broader spectrum of preserved specimens would address the limitations imposed by the current dataset.

Another critical aspect warranting further evaluation is the usability of deep learning-based applications across diverse biodiversity datasets. Research by Campos et al. (2024), Campos et al. (2023) has demonstrated that the efficacy of artificial intelligence algorithms in species identification can vary significantly depending on the animal dataset. This variability underscores the potential utility of applications, such as the one proposed in this study, as supportive technologies for field experts rather than as standalone solutions intended to replace human expertise.

Overall, this study underscores the necessity for further development and investigation of reliable models for biodiversity monitoring and research, with particular emphasis on endemic Amazonian lizards. The promising results presented herein pave the way for future advancements in this critical area of conservation science.

## Data Availability

The raw data supporting the conclusions of this article will be made available by the authors, without undue reservation.

## References

[ref1] Abadi M. Agarwal A. Barham P. Brevdo E. Chen Z. Citro C.. (2016). Tensor flow: large-scale machine learning on heterogeneous distributed systems. arXiv.

[ref2] Bell T. P. (2009). A novel technique for monitoring highly cryptic lizard species in forests. Herpetol. Conserv. Biol. 4, 415–425.

[ref3] Binta Islam S. Valles D. Hibbitts T. J. Ryberg W. A. Walkup D. K. Forstner M. R. J. (2023). Animal species recognition with deep convolutional neural networks from ecological camera trap images. Animals 13:1526. doi: 10.3390/ani13091526, PMID: 37174563 PMC10177479

[ref4] Bolon I. Picek L. Durso A. M. Alcoba G. Chappuis F. Ruiz De Castañeda R. (2022). An artificial intelligence model to identify snakes from across the world: opportunities and challenges for Global Health and herpetology. SSRN Electron. J. 16, e0010647. doi: 10.1371/journal.pntd.0010647 PMC9426939 35969634

[ref5] Campos D. S. de Oliveira R. F. Oliveira Vieira L. de Bragança P. H. N. Guimarães E. C. Katz A. M. (2024). Well-intentioned initiatives hinder understanding biodiversity conservation: an essay on a recent deep-learning image classifier for Amazonian fishes Rev. Fish Biol. Fish. 187–200 doi: 10.1007/s11160-024-09901-y 35

[ref6] Campos D. S. Oliveira R. F. D. Vieira L. D. O. Bragança P. H. N. D. Nunes J. L. S. Guimarães E. C.. (2023). Revisiting the debate: documenting biodiversity in the age of digital and artificially generated images. Web Ecol. 23, 135–144. doi: 10.5194/we-23-135-2023

[ref7] Chen R. Little R. Mihaylova L. Delahay R. Cox R. (2019). Wildlife surveillance using deep learning methods. Ecol. Evol. 9, 9453–9466. doi: 10.1002/ece3.5410, PMID: 31534668 PMC6745675

[ref8] Da Costa Prudente A. L. Ramos L. Silva T. Sarmento J. Dourado A. Silva F.. (2019). Dataset from the snakes (Serpentes, reptiles) collection of the Museu Paraense Emílio Goeldi, Pará, Brazil. Biodivers Data J. 7:e34013. doi: 10.3897/BDJ.7.e34013, PMID: 31086477 PMC6483954

[ref9] Durso A. M. Moorthy G. K. Mohanty S. P. Bolon I. Salathé M. Ruiz De Castañeda R. (2021). Supervised learning computer vision benchmark for Snake species identification from photographs: implications for herpetology and Global Health. Front Artif Intell. 4:582110. doi: 10.3389/frai.2021.582110, PMID: 33959704 PMC8093445

[ref10] Frazier P. I. (2018). A tutorial on Bayesian optimization. arXiv.

[ref11] Gill K. S. Gupta R Malhotra S Devliyal S Sunil G (2024). Classification of reptiles and amphibians using transfer learning and deep convolutional neural networks. In: 2024 IEEE 9th International Conference for Convergence in Technology (I2CT) [Internet]. Pune, India: IEEE. pp. 1–5. Available at: https://ieeexplore.ieee.org/document/10544030/ (Accessed November 2, 2024).

[ref12] Gomez Villa A. Salazar A. Vargas F. (2017). Towards automatic wild animal monitoring: identification of animal species in camera-trap images using very deep convolutional neural networks. Ecol. Inform. 41, 24–32. doi: 10.1016/j.ecoinf.2017.07.004

[ref9001] Gray P. C. Bierlich K. C. Mantell S. A. Friedlaender A. S. Goldbogen J. A. Johnston D. W. (2019). Drones and convolutional neural networks facilitate automated and accurate cetacean species identification and photogrammetry. Methods Ecol Evol. 10, 1490–1500.

[ref13] Haq A. U. Zhang D. Peng H. Rahman S. U. (2019). Combining multiple feature-ranking techniques and clustering of variables for feature selection. IEEE Access 7, 151482–151492. doi: 10.1109/ACCESS.2019.2947701

[ref14] Howard A. Sandler M. Chu G. Chen L. C. Chen B. Tan M.. (2019). Searching for MobileNetV3. arXiv.

[ref15] Howard A. G. Zhu M. Chen B. Kalenichenko D. Wang W. Weyand T.. (2017). Mobile nets: efficient convolutional neural networks for mobile vision applications. arXiv.

[ref16] Islam SB Valles D (2020). “Identification of wild species in Texas from camera-trap images using deep neural network for conservation monitoring.” In: *2020 10th Annual Computing and Communication Workshop and Conference (CCWC)*. Las Vegas, NV, USA: IEEE. pp. 0537–0542.

[ref17] Jovel J. Greiner R. (2021). An introduction to machine learning approaches for biomedical research. Front. Med. 8:771607. doi: 10.3389/fmed.2021.771607, PMID: 34977072 PMC8716730

[ref18] Kornblith S Shlens J Le QV (2019). “Do Better ImageNet Models Transfer Better?” in *2019 IEEE/CVF Conference on Computer Vision and Pattern Recognition (CVPR) [internet]*. Long Beach, CA, USA: IEEE. pp. 2656–2666.

[ref19] Martineau C. Conte D. Raveaux R. Arnault I. Munier D. Venturini G. (2017). A survey on image-based insect classification. Pattern Recogn. 65, 273–284. doi: 10.1016/j.patcog.2016.12.020

[ref20] Mäyrä J. Keski-Saari S. Kivinen S. Tanhuanpää T. Hurskainen P. Kullberg P.. (2021). Tree species classification from airborne hyperspectral and LiDAR data using 3D convolutional neural networks. Remote Sens. Environ. 256:112322. doi: 10.1016/j.rse.2021.112322

[ref21] Miao Z. Gaynor K. M. Wang J. Liu Z. Muellerklein O. Norouzzadeh M. S.. (2019). Insights and approaches using deep learning to classify wildlife. Sci. Rep. 9:8137. doi: 10.1038/s41598-019-44565-w, PMID: 31148564 PMC6544615

[ref22] Michele A. Colin V. Santika D. D. (2019). Mobilenet convolutional neural networks and support vector machines for palmprint recognition. Procedia Comput. Sci. 157, 110–117. doi: 10.1016/j.procs.2019.08.147

[ref23] Murphy J. C. Lehtinen R. M. Charles S. P. Wasserman D. Anton T. Brennan P. J. (2017). Cryptic multicolored lizards in the *Polychrus marmoratus* group (Squamata: Sauria: Polychrotidae) and the status of *Leiolepis auduboni* Hallowell. Amphib. Reptile. 11, 1–16.

[ref24] Naser M. Z. Alavi A. (2023). Insights into performance fitness and error metrics for machine learning. Archit. Struct. Constr. 3, 499–517.

[ref25] Nezami S. Khoramshahi E. Nevalainen O. Pölönen I. Honkavaara E. (2020). Tree species classification of drone hyperspectral and RGB imagery with deep learning convolutional neural networks. Remote Sens 12:1070. doi: 10.3390/rs12071070

[ref26] Norouzzadeh M. S. Morris D. Beery S. Joshi N. Jojic N. Clune J. (2021). A deep active learning system for species identification and counting in camera trap images Schofield M, editor. Methods Ecol. Evol. 12, 150–161. doi: 10.1111/2041-210X.13504

[ref27] Pedregosa F. Varoquaux G. Gramfort A. Michel V. Thirion B. (2008). Scikit-learn: machine learning in python. J. Mach. Learn. 9, 2825–2830.

[ref28] Pinho C. Kaliontzopoulou A. Ferreira C. A. Gama J. (2023). Identification of morphologically cryptic species with computer vision models: wall lizards (Squamata: Lacertidae: *Podarcis*) as a case study. Zool. J. Linnean Soc. 198, 184–201. doi: 10.1093/zoolinnean/zlac087

[ref29] Polonen I Annala L Rahkonen S Nevalainen O Honkavaara E Tuominen S (2018). “Tree species identification using 3D spectral data and 3D convolutional neural network.” In: *2018 9th Workshop on Hyperspectral Image and Signal Processing: Evolution in Remote Sensing (WHISPERS)*. Amsterdam, Netherlands: IEEE. pp. 1–5.

[ref30] Pyron R. Burbrink F. T. Wiens J. J. (2013). A phylogeny and revised classification of Squamata, including 4161 species of lizards and snakes. BMC Evol. Biol. 13:93. doi: 10.1186/1471-2148-13-93, PMID: 23627680 PMC3682911

[ref31] Raju VNG Lakshmi KP Jain VM Kalidindi A Padma V (2020). “Study the influence of normalization/transformation process on the accuracy of supervised classification.: In *2020 Third International Conference on Smart Systems and Inventive Technology (ICSSIT)*. Tirunelveli, India: IEEE. pp. 729–735.

[ref32] Robillard A. J. Trizna M. G. Ruiz-Tafur M. Dávila Panduro E. L. Santana C. D. White A. E.. (2023). Application of a deep learning image classifier for identification of Amazonian fishes. Ecol. Evol. 13:e9987. doi: 10.1002/ece3.9987, PMID: 37143991 PMC10151603

[ref33] Rossum GV (1995). Python Tutorial Centrum voor Wiskunde en Informatica Amsterdam. The Netherlands.

[ref34] Sandler M Howard A Zhu M Zhmoginov A Chen LC (2018). “MobileNetV2: inverted residuals and linear bottlenecks.” In: *2018 IEEE/CVF Conference on Computer Vision and Pattern Recognition*. Salt Lake City, UT: IEEE. p. 4510–4520.

[ref35] Sharmin I. Islam N. F. Jahan I. Ahmed Joye T. Rahman M. R. Habib M. T. (2019). Machine vision based local fish recognition. SN Appl. Sci. 1:1529. doi: 10.1007/s42452-019-1568-z

[ref36] Sowmya M. Balasubramanian M. Vaidehi K. (2023). “Classification of animals using MobileNet with SVM classifier” in Computational methods and data engineering. eds. Asari V. K. Singh V. Rajasekaran R. Patel R. B., Lecture Notes on Data Engineering and Communications Technologies; vol. 139 (Singapore: Springer Nature Singapore), 347–358.

[ref37] Stewart G. Daniel R. S. (1075). Microornamentation of lizard scales: some variations and taxonomic correlations. Herpetologica 31, 117–130.

[ref38] Torres-Carvajal O. De Queiroz K. (2009). Phylogeny of hoplocercine lizards (Squamata: Iguania) with estimates of relative divergence times. Mol. Phylogenet. Evol. 50, 31–43. doi: 10.1016/j.ympev.2008.10.002, PMID: 18952184

[ref39] Tuia D. Kellenberger B. Beery S. Costelloe B. R. Zuffi S. Risse B.. (2022). Perspectives in machine learning for wildlife conservation. Nat. Commun. 13:792. doi: 10.1038/s41467-022-27980-y, PMID: 35140206 PMC8828720

[ref40] van der Maaten L. Hinton G. (2008). Visualizing data using t-SNE. J. Mach. Learn. 9, 2579–2605.

[ref41] Vitt L. J. Avila-Pires T. C. S. Zani P. A. Sartorius S. S. Espósito M. C. (2003). Life above ground: ecology of *Anolis fuscoauratus* in the Amazon rain forest, and comparisons with its nearest relatives. Can. J. Zool. 81, 142–156. doi: 10.1139/z02-230

[ref42] Wäldchen J. Mäder P. (2018). Machine learning for image based species identification. Methods Ecol. Evol. 9, 2216–2225.

[ref43] Wang H Van Stein B Emmerich M Back T (2017). “A new acquisition function for Bayesian optimization based on the moment-generating function.” In: *2017 IEEE International Conference on Systems, Man, and Cybernetics (SMC)*. Banff, AB: IEEE. pp. 507–512.

[ref44] Weinstein B. G. (2018). A computer vision for animal ecology. Prugh L, editor. J. Anim. Ecol. 87, 533–545. doi: 10.1111/1365-2656.12780, PMID: 29111567

[ref45] Xu M. Yoon S. Fuentes A. Park D. S. (2023). A comprehensive survey of image augmentation techniques for deep learning. Pattern Recogn. 137:109347. doi: 10.1016/j.patcog.2023.109347

